# Haemocyte-Derived Innate Immune Reactions of the Giant African Snail (*Lissachatina fulica*) Against the Lungworm *Angiostrongylus vasorum*

**DOI:** 10.3390/ani16142150

**Published:** 2026-07-11

**Authors:** Alena Dusch, Iván Conejeros, Zahady D. Velásquez, Carlos Hermosilla, Anja Taubert

**Affiliations:** Institute of Parasitology, Biomedical Research Center Seltersberg (BFS), Justus Liebig University Giessen, 35392 Giessen, Germany

**Keywords:** *Lissachatina fulica*, *Angiostrongylus vasorum*, gastropod-borne diseases, haemocytes, reactive oxygen species

## Abstract

The metastrongyloid parasite *Angiostrongylus vasorum* is currently considered an emerging gastropod-borne lungworm species of canids and has gained growing scientific attention in recent years. Nonetheless, knowledge of gastropod innate immune reactions and how *A. vasorum* larvae are attacked by gastropod-derived haemocytes is still very sparse. The current study aims to investigate gastropod haemocyte innate immune reactions in response to *A. vasorum* L1 larvae and antigen (*Av*Ag) in vitro. Gastropod haemocytes were isolated from the giant African snail (*Lissachatina fulica*) via cardiac puncture, confronted with *Av*Ag or *A. vasorum* L1, and thereafter assessed for cell activation status, ROS production and extrusion of invertebrate extracellular phagocyte traps (InEPT). Haemocyte–parasite interactions were assessed via flow cytometry, live cell 3D-holotomographic microscopy as well as scanning electron microscopy (SEM). Overall *Av*Ag stimulation activated haemocytes and induced a time-dependent increase in haemocyte ROS production. Moreover, SEM analyses illustrated the formation of ‘InEPT-like’ structures in response to *A. vasorum* L1. These novel findings on neglected invertebrate innate immune responses will help to better understand the complex mollusc–metastrongyloid parasite interactions, which contribute to the epizootiology of not only canine angiostrongylosis but also of closely related metastrongyloid nematodes of public and veterinary health concern.

## Simple Summary

Gastropod-borne metastrongyloid lungworms such as *Angiostrongylus vasorum* have become more relevant in recent years. Little is known about early gastropod-derived haemocyte innate immune reactions against *A. vasorum* larvae and their specific antigens. In this study, we assessed haemocyte effector mechanisms such as production of reactive oxygen species (ROS) and the release of extracellular traps against this canid lungworm species. Present findings will contribute to the neglected field of invertebrate immunity and to better understand metastrongyloid–gastropod interactions.

## 1. Introduction

Gastropod-borne diseases, albeit neglected, play an important role in both veterinary and human medicine [1]. As such, aquatic, semi-aquatic and terrestrial gastropods play a pivotal role as obligatory intermediate hosts in the life cycles of numerous helminths, including all parasitic trematodes [2,3,4] and some metastrongyloid nematode species [5,6]. Metastrongyloid lungworms of canids and felids, including the highly pathogenic species *Angiostrongylus vasorum*, are of great veterinary concern worldwide and are nowadays emerging into previously non-endemic geographic areas [7]. Clinical signs of *A. vasorum* infections in dogs and wild canids vary from mild respiratory signs to severe cardiopulmonary disorders, haemorrhaging, in addition to neurological, ocular, and gastrointestinal symptoms [8,9,10,11]. The heteroxenous life cycle of *A. vasorum* is complex since it includes numerous wild canids (e.g., wolves, jackals, coyotes, foxes) and domestic dogs as definitive hosts and a broad spectrum of gastropods serving as intermediate hosts (i.e., slugs, semi-slugs, snails) besides paratenic hosts [12,13,14,15,16]. By feeding on the faeces of an *A. vasorum*-infected definitive host, gastropod intermediate hosts become infected with first-stage larvae (L1). Alternatively, exogenous L1 may penetrate the gastropod’s epidermis for infection [17,18]. While migrating in the gastropod, *A. vasorum* L1 moult into second-stage (L2) and third-stage larvae (L3), the latter of which represents the infective stage for final hosts. Besides *A. vasorum*, zoonotic *Angiostrongylus cantonensis* and *Angiostrongylus costaricensis* have re-emerged in recent years as relevant gastropod-borne human infections, causing severe symptoms like eosinophilic meningitis and abdominal angiostrongyliasis [19,20]. Of note, the invasive terrestrial giant African snail (*Lissachatina fulica*) is a well-known intermediate host of both *A. vasorum* and *A. cantonensis* in the tropics of South America [1,21,22] and therefore of public health concern. Originally native to East Africa, these giant snails are currently recognised as invasive neozoa in multiple tropical and subtropical countries, including Argentina, Brazil, Colombia, Cuba, Ecuador, India, and the United States of America [23,24,25,26,27,28]. This invasive and coprophagic giant snail may also carry non-native pathogens, such as bacteria, viruses, fungi and other parasites, and thus indirectly affect human and animal health. Additionally, *L. fulica* impacts local agriculture by feeding on a large variety of plants and destroying whole harvests [28,29,30]. Besides the snail’s negative impact on humans, animals, agriculture and ecosystems, it may also be useful as a model organism for gastropod-borne diseases, as recently reported [31,32,33].

In contrast to the innate and adaptive branches of the immune system in mammals and birds, invertebrate gastropods exclusively possess an innate immune system, which plays a crucial role in the defence against invasive pathogens, including lungworms [31,34]. Notably, the gastropod innate immune system resembles that reported in vertebrates at the level of cellular and molecular mechanisms [35,36,37]. Gastropods have a cardiopulmonary system and vascularisation, containing haemolymph with haemocytes. Haemocytes are the most abundant cells in the haemolymph, representing the first line of defence and acting as multifunctional professional phagocytes. As such, circulating and tissue-resident haemocytes own various effector mechanisms like phagocytosis, encapsulation, cell-mediated cytotoxicity, and reactive oxygen species (ROS) production, in addition to secretion of microbiocidal and immunomodulatory proteins and enzymes, such as lysozyme, peroxidases, histones and lectins, among others [38,39]. Haemocyte nitric oxide (NO) synthesis represents another critical effector mechanism, since NO not only acts as a signalling molecule but also possesses direct antimicrobial properties [40]. Recent studies also reported on the formation of invertebrate extracellular phagocyte traps (InEPT), which are released from activated gastropod haemocytes in response to the recognition of larval stages of parasitic nematodes like *A. vasorum*, *Aelurostrongylus abstrusus* and *Troglostrongylus brevior* [31,34]. Resembling neutrophil extracellular traps (NETs) in mammals, InEPT release is considered a conserved innate effector mechanism and cell death process directed against large-sized pathogens [31]. Even though it is generally accepted that invertebrates exclusively possess an innate immune system [41,42], some findings also indicate the presence of specialised adaptive immune reactions in these organisms [43,44,45,46,47]. Consistently, invertebrate haemocytes may play a pivotal role in these adaptive immune responses, as suggested elsewhere [43,48]. Hence, gastropod haemocytes regulate cell proliferation, express and release humoral factors [e.g., fibrinogen-related proteins (FREPs), lectins, lysozymes, peroxidases] and own memory functions [48,49].

This study aimed to investigate early gastropod haemocyte-mediated innate immune reactions induced by the lungworm *A. vasorum* by utilising the giant African snail as a suitable model [33]. Here, *L. fulica* haemocytes were stimulated with the *A. vasorum* soluble antigen (*Av*Ag) as well as *A. vasorum* L1 and thereafter assessed for activation, ROS production and InEPT extrusion. Haemocyte–parasite interactions were analysed via flow cytometry, live cell 3D-holotomographic microscopy and scanning electron microscopy (SEM). A novel technique of intracardiac puncture is here presented to obtain considerable haemolymph volumes and haemocyte numbers under standardised in vitro gastropod breeding conditions. Current findings provide novel insights into *L. fulica* haemocyte reactions against *A. vasorum,* which might translationally help to better understand zoonotic-relevant *A. cantonensis* and *A. costaricensis* infections. Taking the increasing popularity of *L. fulica* as pets into account [50,51], raising awareness of the potential health risks associated with handling the snails is essential.

## 2. Materials and Methods

### 2.1. Standardised Gastropod Maintenance

Giant African snails (*L. fulica*) were kept under standardised conditions in a fully automated climate chamber (ECP01E, Snijders Scientific B.V., Tilburg, the Netherlands). Humidity and temperature mimicked the gastropod natural habitat in the tropics by applying temperature ranging from 20 to 26 °C and 50% humidity [52]. The lighting cycle consisted of 10 h of light and 10 h of darkness, with 2 h for dawn and dusk each. Snails were kept in plastic boxes with a 5 cm layer of commercial coconut soil (Kokosfaser–Humusziegel, TropicShop, Nordhorn-Klausheide, Germany) and fed with lettuce (*Lactuca sativa*), carrots (*Daucus carota*), cucumber (*Cucumis sativus*), zucchini (*Cucurbita pepo*), as well as commercial dog food (Premium Trockenfutter Romeo, Mühlheim an der Ruhr, Germany). Common cuttlefish shells (*Sepia officinalis*) (Sepiaschalen, TropicShop, Nordhorn-Klausheide, Germany) were offered ad libitum as a natural calcium source.

### 2.2. Haemolymph Extraction and Haemocyte Isolation

A total of eight adult snails were used for haemolymph extraction. These specimens ranged in age from one to five years. Haemolymph extraction was performed by intracardiac puncture. Briefly, adult *L. fulica* were carefully washed with tap water and dried using paper towels before cardiac puncture. A hole was carefully drilled close to the pneumostome using a commercial drilling instrument (Dremel 3000, Dremel, Robert Bosch Tool Corporation, Racine, WI, USA) until the shell was fully perforated (see Supplementary Video S1). Afterwards, a sterile syringe (23 G) was inserted to puncture the gastropod heart (see Supplementary Video S2). A haemolymph volume of up to one percent of the snail body weight (BW) was extracted (i.e., 500–1000 µL) and immediately mixed 1:1 with an anticoagulant gastropod haemolymph buffer (186 mM NaCl, 98 mM NaOH, 1.7 mM EDTA and 41 mM citric acid, pH 4.5) containing 3% penicillin (500 U/mL; Sigma-Aldrich, Darmstadt, Germany) and streptomycin (500 μg/mL; Sigma-Aldrich) according to Lange et al. [34]. Afterwards, haemocytes present in the haemolymph sample were counted using a Neubauer chamber (Hecht assistent, Sondheim vor der Rhön, Germany). Haemolymph (10 µL) mixed 1:1 with anticoagulant haemolymph buffer was loaded into the Neubauer chamber. The haemocytes present in the four squares of the chamber were counted, and the haemocyte concentration (cells/mL) was calculated. Exemplary haemocyte counts for individual specimens are provided in Supplementary Table S1.

### 2.3. Preparation of Angiostrongylus vasorum L1 Antigen (AvAg)

Vital *A. vasorum* L1 were isolated from the faeces of a naturally infected dog via the Baermann funnel technique. Briefly, 5 g of faeces were placed on a sieve with a 100 µm mesh size. The sieve was mounted on a plastic funnel and the funnel outlet was linked to a plastic tube closed with a metal clamp. The funnel was filled with water, until the faecal material in the sieve was half covered. After 24 h of incubation, the clamp on the tube was carefully opened, and 10 mL of faecal fluid containing viable *A. vasorum* L1 was extracted and washed 3 times with sterile PBS. Afterwards, 1% penicillin (500 U/mL; Sigma-Aldrich) and streptomycin (500 μg/mL; Sigma-Aldrich, Darmstadt, Germany) were added to the sample, which was then stored at 4 °C for 2 days. Larvae were then washed thrice in sterile PBS and homogenised via a sonifier (Branson Ultrasonics SFX150, Branson Ultrasonics, Brookfield, CT, USA). A 60% pulse force was applied for one minute, during which the samples were kept on ice. Afterwards, the samples were centrifuged at 1300 rpm for 5 min at 4 °C. The pellet was discarded, and the soluble *Av*Ag (supernatant) was stored at −80 °C. The protein concentration was measured using the Pierce BCA Protein Assay Kit (ThermoFisher, Waltham, MA, USA). Briefly, the BCA working reagent was prepared by mixing 50 parts of BCA reagent A with 1 part of BCA reagent B (50:1). A total of 5 µL of a BSA standard or soluble *Av*Ag was added into a 96-well plate. Subsequently, 200 µL of working reagent was added to each well and mixed thoroughly by pipetting. Afterwards, the plate was covered and incubated at 37 °C for 30 min before being cooled down to room temperature (RT) for 5 min. Absorbance at 562 nm was measured using a Varioskan microplate reader (ThermoFisher). Protein concentrations were calculated from a BSA standard curve and corrected for the dilution factors. Samples were pooled and adjusted to a final concentration of 1 µg/µL.

### 2.4. Quantification of Reactive Oxygen Species (ROS) by Flow Cytometry

For ROS detection, haemocytes were loaded with 10 µM DCFH-DA (2,7-dichlorodihydrofluorescein diacetate) for 15 min at 22 °C. Unloaded haemocytes served as controls for autofluorescence and background fluorescence signal detection. To assess the effect of *Av*Ag stimulation on haemocyte ROS production, 1 × 10^3^ haemocytes were stimulated for 5, 60 and 120 min with soluble *Av*Ag (1, 10, 100, and 1000 µg/mL) and the fluorescence intensity (FL1) was thereafter registered at RT. For positive and negative controls, haemocytes were stimulated for the same time with the calcium ionophore A23187 5μM (Sigma-Aldrich, Germany) or left in RPMI medium, respectively. Data acquisition was performed using a BD Accuri C6^®^ plus flow cytometer (BD Biosciences, Heidelberg, Germany), analysing approximately 400 cells per time point and experimental condition. After the corresponding gating, the percentage of DCFH-DA^+^ cells was determined.

### 2.5. Live Cell 3D-Holotomography

For live cell 3D-holotomography, isolated haemocytes (*n* = 2) in sterile PBS, pH 7.4, were placed into a 35 mm imaging dish plate (Ibidi^®^, Martinsried, Germany) in a top-stage incubator (Ibidi^®^, Martinsried, Germany). Refractive index (RI)-based 3D-holotomographic images were obtained by using a 3D Cell Explorer-Fluo^®^ (Nanolive, Tolochenaz, Switzerland) microscope equipped with a ×60 magnification (λ = 520 nm, sample exposure 0.2 mW/mm^2^) and a field depth of 30 µm. Images were captured and analysed using the device-specific STEVE^®^ software v.1.6.3496 (Nanolive, Switzerland) to obtain an RI-based z-stack. All images are displayed as maximum z-projections, and gamma, brightness, and contrast were adjusted (identically for compared image sets) using Fiji software (ImageJ, version 2.16.0).

### 2.6. Scanning Electron Microscopy (SEM)

Isolated haemocytes were left in RPMI medium or stimulated for 60 min at RT with LPS (0.1 ng/µL; Sigma-Aldrich) for negative and positive controls, respectively, and exposed to *A. vasorum* L1 and *A. vasorum* L1 antigen (*Av*Ag, 10 µg/mL). For fixation, 2.5% glutaraldehyde (Merck; Darmstadt, Germany) was used. Samples were washed in distilled water, dehydrated, critical point dried by CO_2_ treatment, and finally sputtered with gold. Afterwards, the samples were analysed by a scanning electron microscope (Philips XL30, Eindhoven, The Netherlands) at the Institute of Anatomy and Cell Biology, Justus Liebig University, Giessen, Germany.

## 3. Results

### 3.1. Visualisation of Haemocyte Behaviour via Live Cell 3D-Holotomography

**Figure 1:**
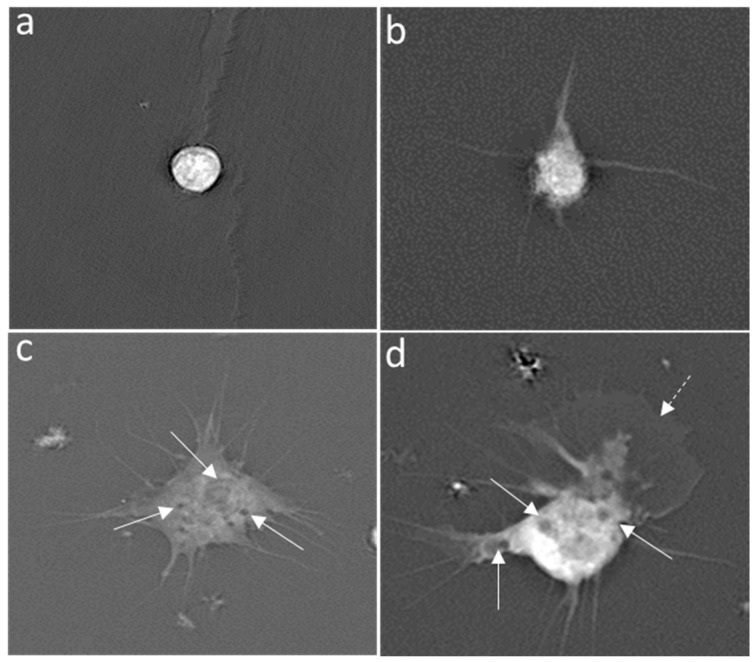
*Lissachatina fulica* haemocytes during different stages of asynchronous and spontaneous cell activation. (**a**) Inactive round haemocyte with flat membrane surface; (**b**) start of cell activation with attachment and pseudopod formation; (**c**,**d**) fully activated, firmly attached haemocytes with pseudopod formation containing granules (solid arrows), veil-like extensions (dashed arrow).

To characterise haemocyte baseline morphology and behaviour, we analysed the cells unstimulated by antigen or larvae. Haemocyte morphology changed asynchronously and spontaneously, with different morphological cell changes appearing at different stages of the response (Figure 1). At the early stages of culture, the formation of a few pseudopodia and an increased cell mobility and attachment was observed (Figure 1b), whereas at later stages, haemocytes showed extensive restructuring of the cytosol, the increased visibility of intracellular granules of various sizes and larger pseudopodia arrangements (Figure 1c,d). In some instances, we observed that pseudopodia from neighbouring haemocytes appeared to merge, thereby forming larger structures. In addition, broad veil-like extensions attached to the substrate were illustrated in multiple activated haemocytes (Figure 1d).

### 3.2. Exposure of Haemocytes to A. vasorum L1 Drives the Formation of Invertebrate Extracellular Phagocyte Trap ‘(InEPT)-like’ Structures

**Figure 2:**
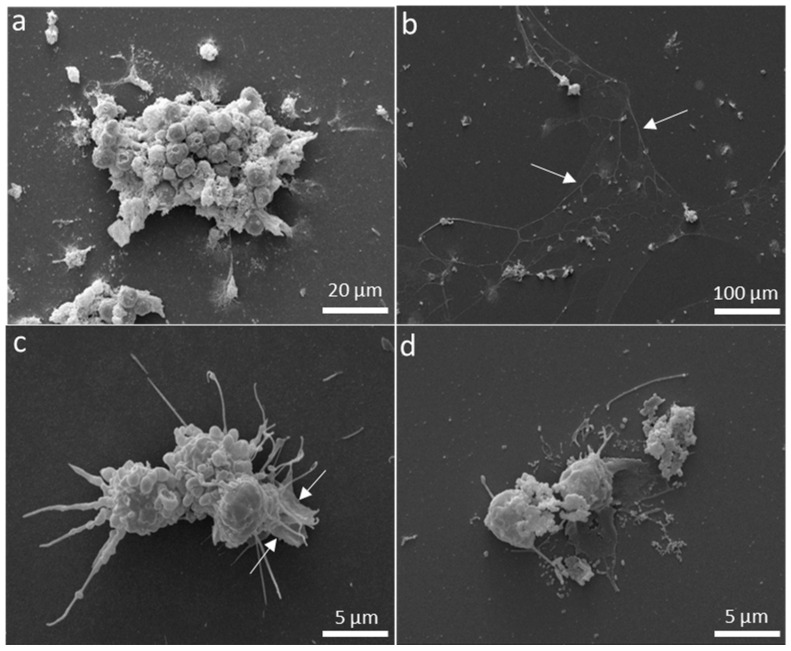
SEM images of *Lissachatina fulica* haemocytes reacting to different stimuli. *Angiostrongylus vasorum* L1 induced (**a**) haemocyte aggregation, (**b**) the formation of ‘spread InEPT-like’ (‘*spr*InEPT’) phenotypes (indicated by white arrows); (**c**) *Av*Ag (10 µg/mL)-induced haemocyte pseudopod formation and irregular surfaces with membrane blebbing and veil-like structures (indicated by white arrows); (**d**) LPS-stimulation-mediated haemocyte activation with cell attachment, ‘*diff*InEPT’-like structures and pseudopod formation.

SEM analysis illustrated phenotypic changes of haemocytes in response to LPS (0.1 ng/µL), *A vasorum* L1 and soluble *Av*Ag, respectively (Figure 2). After the stimulation of haemocytes with multiple *A. vasorum* L1, the formation of haemocyte clusters (Figure 2a) and of both ‘spread InEPT-like’ (‘*spr*InEPT’) and ‘diffuse InEPT-like’ (*‘diff*InEPT’) (Figure 2b) phenotypes was observed, independent of direct contact with the larvae. Moreover, *Av*Ag stimulation led to the formation of haemocyte aggregates with surface blebbing and to enhanced pseudopod formation. In some areas, veil-like structures attaching to the substrate were observed as well (Figure 2c). After LPS stimulation haemocytes were activated, attached to the substrate, formed pseudopodia and extruded fine ‘*diff*InEPT’-like structures (Figure 2d).

### 3.3. AvAg Stimulation Induces ROS Generation in Gastropod Haemocytes

**Figure 3:**
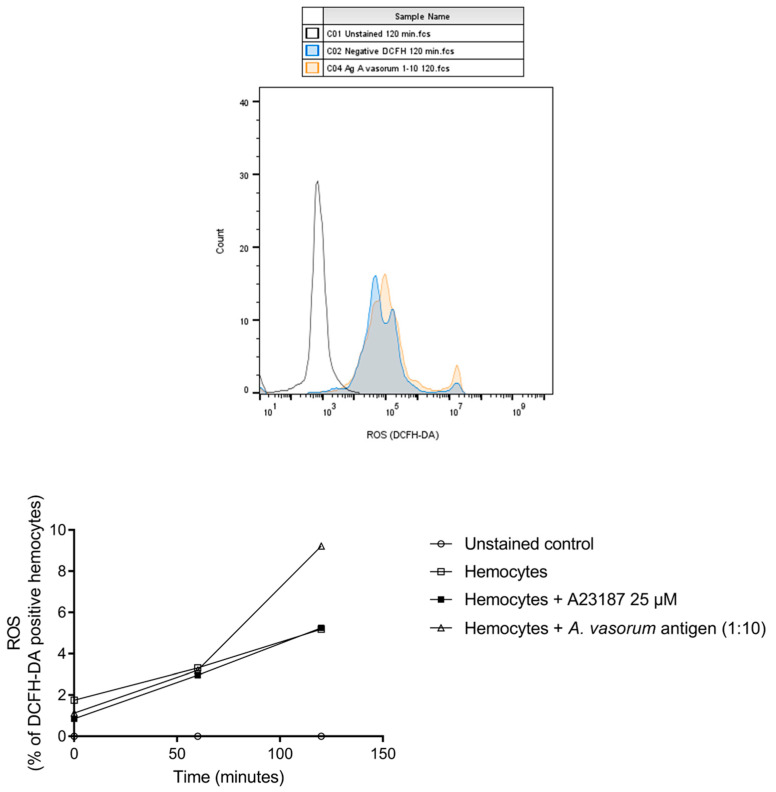
*Angiostrongylus vasorum* antigen (*Av*Ag) stimulates intracellular ROS production by *Lissachatina fulica* haemocytes. For ROS detection, haemocytes were loaded with 10 µM DCFH-DA (2,7-dichlorodihydrofluorescein diacetate) for 15 min at 22 °C. Unstained haemocytes served as controls for autofluorescence and background signal detection. To assess the effect of *Av*Ag on ROS production, 1 × 10^3^ haemocytes were stimulated with soluble *Av*Ag (100 µg/mL) and the fluorescence intensity (FL1) was registered at 60 and 120 min of incubation at RT. For controls, haemocytes were stimulated for the same time with the calcium ionophore A23187 5 μM or left in plain medium. Data acquisition was performed using a BD Accuri C6^®^ plus flow cytometer, analysing approximately 400 cells per time point and experimental condition. After gating, the percentage of DCFH-DA^+^ cells was determined.

To assess inherent oxidative responses, gastropod haemocytes were stimulated for 5, 60 and 120 min with *Av*Ag and tested for intracellular ROS generation via flow cytometry (for gating strategy and single histograms, see Supplementary Figure S1). Here, haemocytes showed time- and antigen concentration-dependent reactions. Hence, an *Av*Ag antigen concentration of 1000 µg/mL was cytotoxic for haemocytes, since it resulted in cell fragmentation. A concentration of 1 and 10 µg *Av*Ag /mL did not yield a clear and consistent effect. At a concentration of 100 µg *Av*Ag /mL, haemocytes responded with increased ROS production after 120 min of exposure when compared to medium controls (Figure 3). Notably, haemocyte stimulation with the calcium ionophore A23187 (5 µM), commonly used as ROS inducers in the mammalian system, did not induce any changes in ROS production (Figure 3).

## 4. Discussion

A broad range of gastropod species act as obligatory intermediate hosts of the highly pathogenic vascular canid nematode *A. vasorum*, which may cause life-threatening disease in dogs. So far, little is known about innate immune reactions generated by *A. vasorum*-infected gastropods. In the current study, we showed that gastropod haemocytes indeed responded to *Av*Ag and *A. vasorum* L1 stimulation by upregulating both the formation of ‘InEPT-like’ structures and intracellular ROS production. ROS synthesis and release by innate immune cells is a well-conserved effector mechanism, widely utilised by professional phagocytes among the animal kingdom, and which has already been described before for other invertebrate species, including the fruit fly *Drosophila* sp. [53], oysters or other bivalves [54,55] and the freshwater snail *Biomphalaria glabrata* [56]. However, to the best of our knowledge, findings on ROS production by *L. fulica* haemocytes are scarce [57], and data on nematode-driven reactions or on ROS dynamics in this mollusc species remain limited. Hence, present findings expand current knowledge on this effector mechanism in *L. fulica* haemocytes within the neglected field of invertebrate immunology and related effector molecules. 

Notably, the stimulation of haemocytes with soluble *Av*Ag (100 µg/mL) drove ROS generation, highlighting the versatility of haemocyte ROS production and confirming these effector molecules as a general defence mechanism against various stimuli, as previously reported for various organisms, including mammals, fish, insects and plants [58,59]. Likewise, oxidative burst activities generally play a key role in the innate immune system of gastropods, defending the organism against various pathogens, such as parasites and bacteria [60,61]. Notably, the adverse effects of ROS on nematode viability have been demonstrated in other host contexts [62].

The formation of extracellular traps (ETs) is a well-documented effector mechanism of mammalian phagocytes utilised in the innate defence against nematode parasites [63,64,65]. A prerequisite for both ET formation and ROS production is the activation of phagocytes. In line with this, SEM analyses phenotypically illustrated haemocyte activation after exposure to all *A. vasorum* L1, soluble *Av*Ag and LPS. Hence, in all cases, the haemocyte surface changed from smooth to rough and the cells extended their membranes by forming pseudopodia. The presence of ‘InEPT-like’ formations in the absence of direct contact with *A. vasorum* L1 suggests that the formation of these ‘InEPT-like’ structures occurred as a response to excretory/secretory products present in the well. Moreover, haemocyte aggregation was here recorded. Notably, and as documented by live cell 3D-holotomography, haemocytes also showed spontaneous activation after ex vivo extraction, but at a minor level. This basal activity likely reflects an inherent patrolling and phagocytic readiness but may also have been driven by mechanical irritation due to the isolation process, such as the extraction itself, the mixing with the anticoagulant buffer and the contact with glass trays. In addition, *L. fulica* snails may encounter some microorganisms even under current standardised maintenance, like bacteria brought into the enclosure with food or soil. Furthermore, the current procedure of haemolymph extraction by cardiac puncture may not be entirely sterile, even though sterile syringes and anticoagulant buffer with antibiotics were used, especially when considering bacteria from the natural microbiome of invertebrate haemolymph [66,67]. However, since only a small proportion of ex vivo-extracted haemocytes showed spontaneous activation, we consider the current isolation protocol as suitable. Moreover, as illustrated in the Supplementary Videos, the procedure of haemolymph extraction also proved to be mild and non-irritating for snail individuals, not at all affecting their well-being and viability (please see Supplementary Materials).

SEM analysis enabled a detailed illustration of ‘InEPT-like’ formations by activated haemocytes. Thin and elongated fibres forming extracellular ‘net-like’ DNA structures in response to *A. vasorum* L1 were observed. Morphological heterogeneity was evident, with different ‘InEPT-like’ structures being documented, i.e., ‘*spr*InEPT’- and ‘*diff*InEPT’-like structures in response to haemocyte *A. vasorum* L1 stimulation. Whether these parasite-induced extracellular structures own the same immunomodulatory properties as mammalian NETs (e.g. anti-inflammatory properties of *agg*NETs [68,69]) awaits further investigations. While the observed structures indeed appear ‘InEPT-like’ based on their morphology, further studies employing molecular assays are essential for definitive verification. Finally, holotomography further complemented conventional microscopic techniques by enabling 3D live cell imaging of haemocytes under almost physiological conditions, i.e., without stressing haemocytes through staining or other experimental procedures, potentially resulting in artificial cell activation, as previously reported [70,71].

While haemocyte reactions were observed against stimuli in vitro, it is essential to recognise that in natural infections, the gastropod’s immune system might respond differently to lungworm infection. Varying factors include a higher or lower dose of larvae infection, the lungworm species itself and/or the gastropod species. It is also unknown whether those innate immune reactions will provide a full immunity and kill the invading larvae. Notably, an *Av*Ag concentration of 1000 µg/mL proved to be cytotoxic to haemocytes, which might suggest that haemocytes are unable to effectively fight a high infectious dose of larvae.

However, several limitations must be considered in the present haemocyte study. Firstly, the number of haemocytes isolated per *L. fulica* specimen is still low and not at all comparable with the mammalian system, thereby hampering extensive analyses on molecular mechanisms, signalling cascades, metabolic signatures, receptor analyses and InEPT formation. Secondly, SEM analyses exclusively provide ultrastructural information on the haemocyte surface but do not allow conclusions on molecular composition or expression of receptors. Moreover, so far, the actual impact of ‘InEPT-like’ structures on invading lungworm larvae remains unclear. However, an excessive InEPT formation may also harm gastropod tissues as reported for mammalian ETs [72,73]. Thirdly, the currently used method for haemocyte isolation from haemolymph does not apply physiological pH and oxygen concentrations, which both were reported to affect innate leukocyte biology [74,75,76]. Future experimentation will pursue obtaining higher numbers of haemocytes for more physiological experimental conditions to better understand gastropod-mediated innate immunity. Finally, this study was designed as a ‘proof of concept’ to assess the feasibility of our research approach, and furthermore to improve gastropod haemolymph extraction by non-molesting animal methods. Therefore, a higher number of individual specimens should be used in future studies to assess possible inter-individual varieties, include studies on pathogen-recognition receptors as well as the molecular characterisation of ‘InEPT-like’ structures.

## 5. Conclusions

In conclusion, here we demonstrate that *A. vasorum* L1 and *Av*Ag exposure to haemocytes leads to the activation of these gastropod phagocytes, accompanied by both ROS production and ‘InEPT-like’ formation. Overall, this work deepens the understanding of gastropod–parasite interactions and of the gastropod innate immune system in general. In addition, these findings highlight *L. fulica* as a promising model organism for studying invertebrate innate immune reactions not only against invading parasites but also other pathogens like bacteria or viruses. Further malacological research is needed to study the complex innate immune system and its impact on the transmission of gastropod-borne diseases worldwide.

## Data Availability

The original contributions presented in this study are included in the article/Supplementary Materials. Further inquiries can be directed to the corresponding author.

## Supplementary Materials

The following supporting information can be downloaded at https://www.mdpi.com/article/10.3390/ani16142150/s1, Video S1: Perforation of *L. fulica* shell above the heart; Video S2: Cardiac puncture and haemolymph extraction from *L. fulica*; Figure S1: Gating strategy and histograms. Table S1: Exemplary haemocyte counts in *Lissachatina fulica*.
