# Supplementary material for: Haemocyte-Derived Innate Immune Reactions of the Giant African Snail (Lissachatina fulica) Against the Lungworm Angiostrongylus vasorum
Source: Animals (Basel). 2026 Jul 11;16(14):2150. doi: 10.3390/ani16142150 (PMC13405181; doi:10.3390/ani16142150)
Supplement: Supplementary file 1 [file animals-16-02150-s001.zip › Supplementary.pptx]

## Slide 1
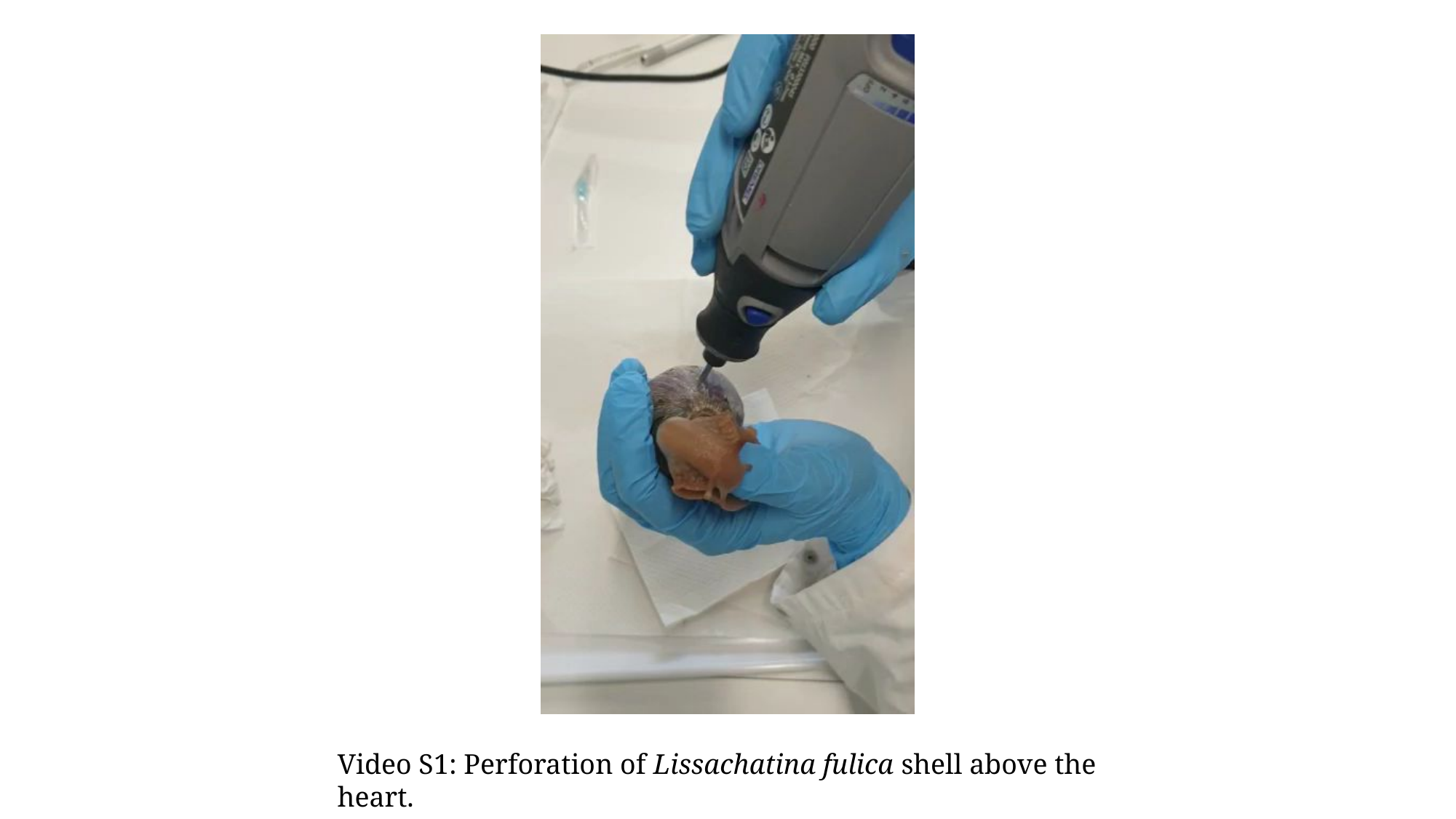

Video S1: Perforation of Lissachatina fulica shell above the heart.

## Slide 2
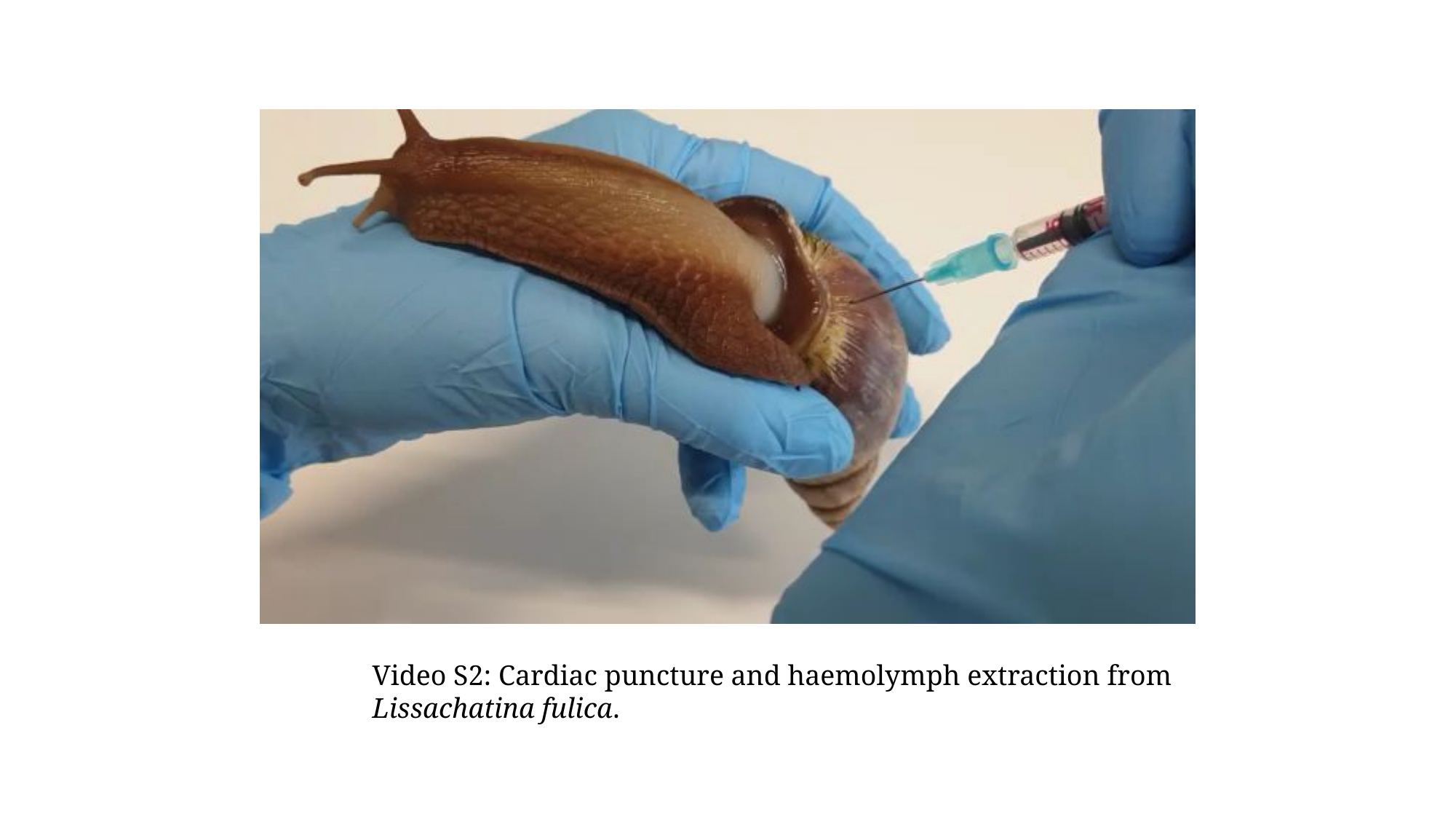

Video S2: Cardiac puncture and haemolymph extraction from Lissachatina fulica.

## Slide 3
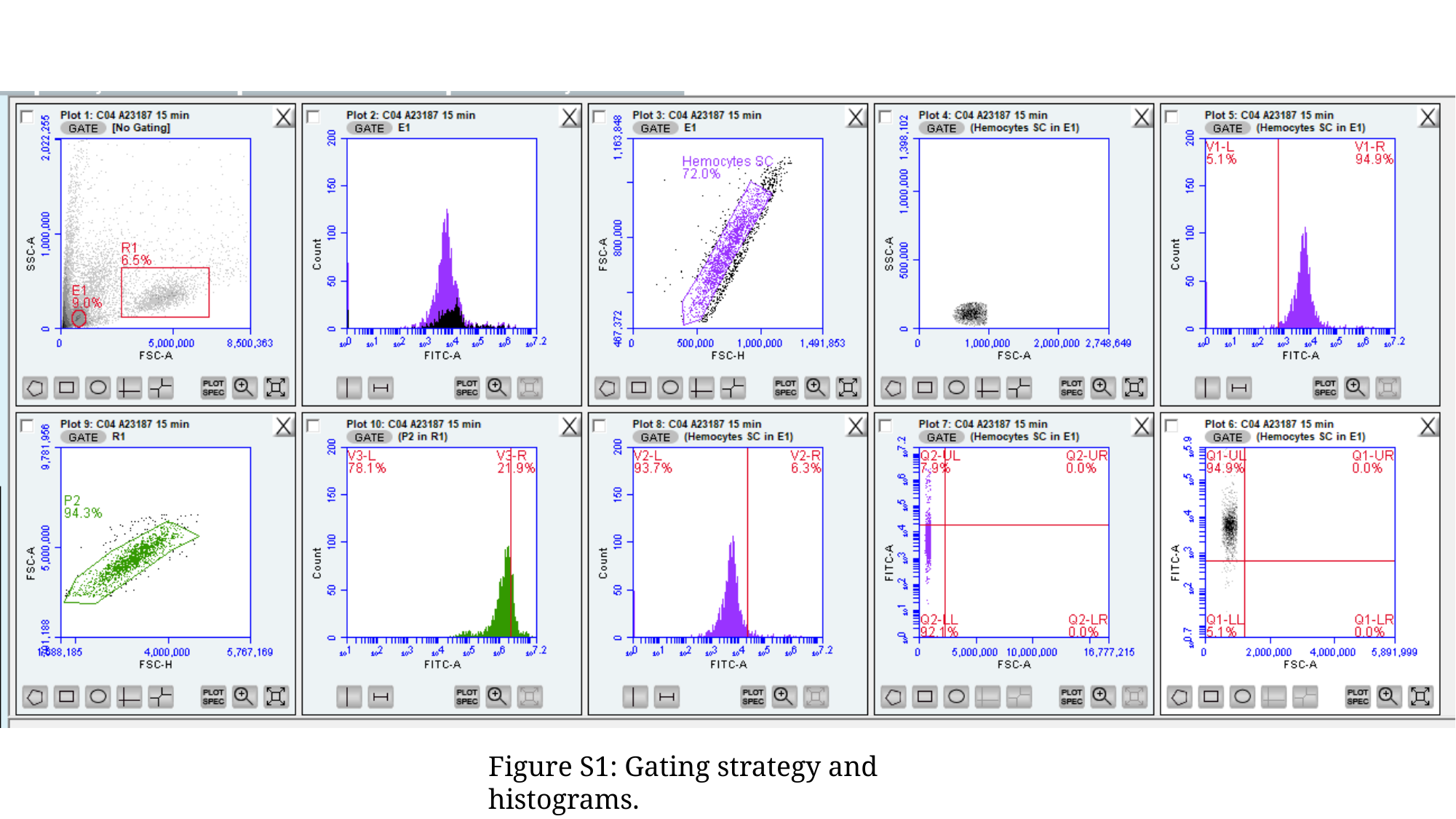

Figure S1: Gating strategy and histograms.

## Slide 4
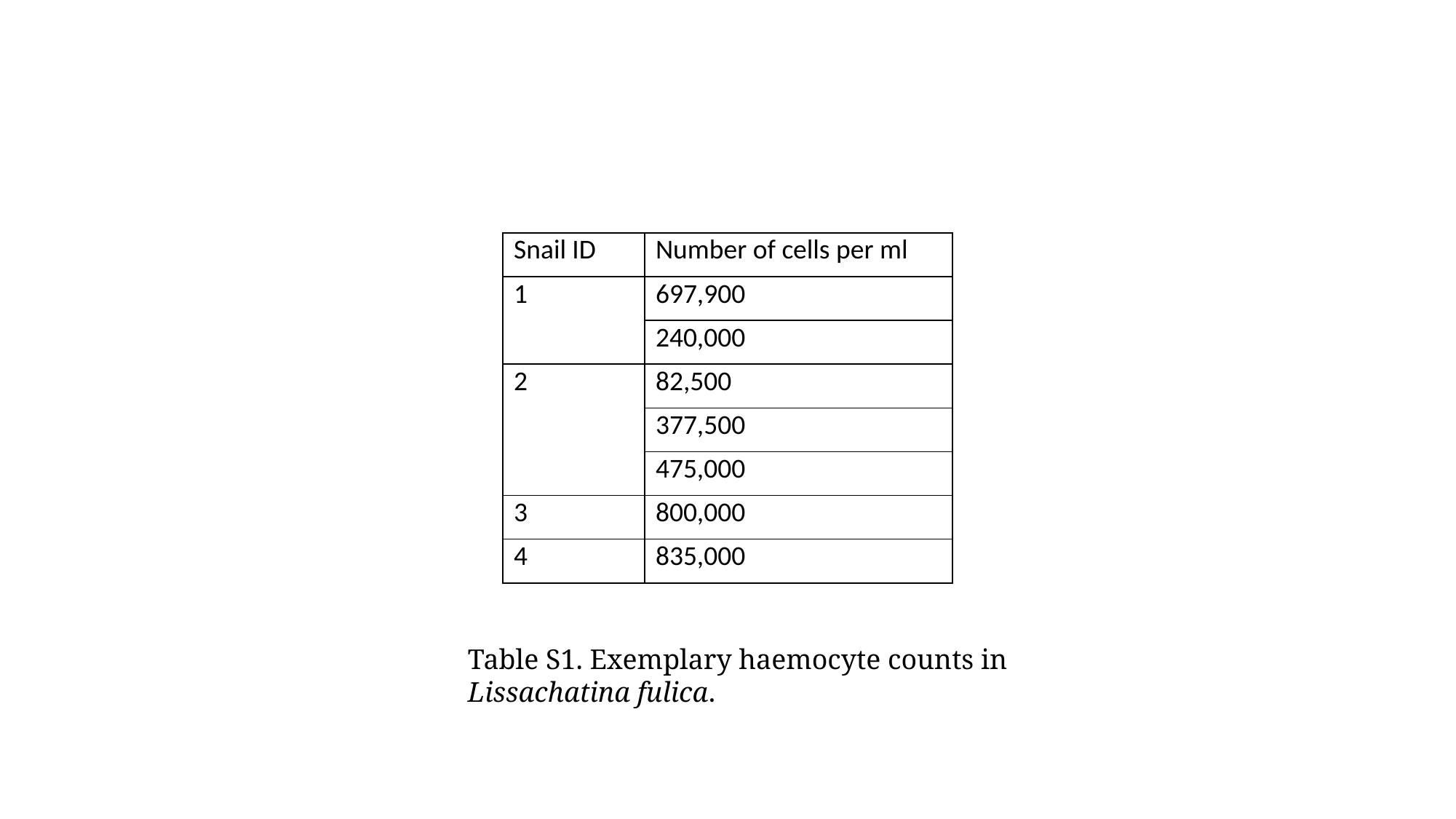

| Snail ID | Number of cells per ml |
| --- | --- |
| 1 | 697,900 |
| | 240,000 |
| 2 | 82,500 |
| | 377,500 |
| | 475,000 |
| 3 | 800,000 |
| 4 | 835,000 |
Table S1. Exemplary haemocyte counts in Lissachatina fulica.
